# In vitro and in vivo investigation of chlorophyll binding sites involved in non‐photochemical quenching in Chlamydomonas reinhardtii

**DOI:** 10.1111/pce.13566

**Published:** 2019-05-09

**Authors:** Federico Perozeni, Stefano Cazzaniga, Matteo Ballottari

**Affiliations:** ^1^ Department of Biotechnology University of Verona Verona Italy

**Keywords:** abiotic stress, *Chlamydomonas*, light harvesting, mutagenesi, photoprotection, photosynthesis

## Abstract

Non‐photochemical quenching (NPQ) of the light energy absorbed is one of the main photoprotective mechanisms evolved by oxygenic photosynthetic organisms to avoid photodamage, at a cost of reduced photosynthetic efficiency. Tuning of NPQ has been reported as a promising biotechnological strategy to increase productivity in both higher plants and unicellular microalgae. Engineering of NPQ induction requires the comprehension of its molecular mechanism(s), strongly debated in the last three decades with several different models proposed. In this work, the molecular details of NPQ induction was investigated at intramolecular level by in vitro and in vitro site‐specific mutagenesis on chlorophyll binding sites of the Light‐Harvesting Complex Stress‐Related 3 (LHCSR3) protein, the pigment binding complexes identified as the quencher during NPQ induction in the model organism for green algae Chlamydomonas reinhardtii. The results obtained demonstrate a correlation between the quenching activity of LHCSR3 variants in vitro and the NPQ phenotypes observed in vivo. In particular, multiple quenching sites in LHCSR3 cooperatively dissipating the excitation energy were revealed with a peculiar role of Chl 613, a chromophore located a close distance to carotenoid binding site L1.

## Abstract 2

Non‐photochemical quenching is a short‐term photoprotective mechanism influencing biomass productivity in phototrophs induced in Chlamydomonas reinhardtii by LHCSR3. Here, site‐specific mutagenesis on LHCSR3 chlorophyll binding sites reveals multiple quenching sites with a peculiar role of Chl 613.

## 1 INTRODUCTION

Plants and algae in their natural environment are constantly exposed to rapid change in quality and intensity of light. When light is absorbed in excess, compared with the metabolic capacity to regenerate ADP and NADP^+^, the precursor of ATP and NADPH, the photosynthetic apparatus incurs in overexcitation. In this conditions, excitation energy at the level of photosystems cannot be properly used for the photochemical process, increasing the probability of energy transfer to oxygen‐forming toxic reactive oxygen species (ROS; Niyogi, 1999). To prevent photodamage caused by ROS formation, a wide range of photoprotective mechanisms have been developed by oxygenic photosynthetic organisms (Niyogi, 1999). Non‐photochemical quenching (NPQ) is the most rapid one and leads to thermal dissipation of excitation energy in a non‐radiative process (Belgio et al., 2014; DemmigAdams et al., 1996; Niyogi, 1999; A. V. Ruban, Johnson, & Duffy, 2012). NPQ has been observed and measured in several organisms, from cyanobacteria (Wilson et al., 2006) to green algae (Niyogi, Bjorkman, & Grossman, 1997; Peers et al., 2009), diatoms (Olaizola, La Roche, Kolber, & Falkowski, 1994; A. Ruban et al., 2004), mosses (Alboresi, Gerotto, Giacometti, Bassi, & Morosinotto, 2010), and higher plants (Demmig‐Adams & Adams, 2000; Li et al., 2000; Niyogi, 1999), being activated rapidly in seconds to minutes time scale with a feedback mechanism triggered by lumen acidification (Ballottari et al., 2016; Li et al., 2004; Liguori, Roy, Opacic, Durand, & Croce, 2013; Peers et al., 2009; Rees et al., 1992). However, activation of non‐radiative dissipative channel of excitation energy increases photoprotection properties at a cost of reduced photosynthetic efficiency: tuning of NPQ induction for a proper balance of photoprotection and photosynthetic efficiency has been reported as a strategy to increase biomass productivity in both higher plants (Kromdijk et al., 2016) and microalgae (Berteotti, Ballottari, & Bassi, 2016). The molecular details of NPQ induction in the different organisms have been extremely debated in the last three decades. Characterization of mutants selected for having an NPQ phenotype, allowed to identify light harvesting complexes (LHC) as the main quenching site during NPQ induction in eukaryotic photosynthetic organisms, with PSBS and LHCSR subunits as NPQ triggers, being able to sense the lumenal pH (Li et al., 2000; Niyogi et al., 1997; Niyogi, Grossman, & Björkman, 1998; Peers et al., 2009). In higher plants, NPQ induction is indeed activated by the thylakoid subunit PSBS, with a minor role of zeaxanthin accumulation upon high light exposure (Li et al., 2000; Niyogi et al., 1998). In the model organism for green algae, C. reinhardtii LHCSR1 and LHCSR3 proteins have been reported to be essential for NPQ induction, whereas the role of xanthophyll cycle is negligible, if any (Bonente et al., 2011; Dinc et al., 2016; Kim, Akimoto, Tokutsu, Yokono, & Minagawa, 2017; Kosuge et al., 2018; Peers et al., 2009). PSBS and LHCSR are present in the thylakoid membranes with protonatable residues exposed to the lumen side (Ballottari et al., 2016; Li et al., 2004; Liguori et al., 2013), being able to sense the lumenal pH and activate NPQ upon acidification. However, the molecular mechanism by which these two proteins induce NPQ is different because PSBS is essentially a membrane subunit that does not bind pigments, whereas LHCSR subunits does (Bonente et al., 2011; Fan et al., 2015). PSBS is thus considered to induce other LHC proteins to switch to a dissipative state (Correa‐Galvis, Poschmann, Melzer, Stühler, & Jahns, 2016; C. Liu et al., 2016; Ware, Giovagnetti, Belgio, & Ruban, 2015), whereas LHCSR subunits have been reported to be directly able to switch to dissipative state at low pH, being at the same time a sensor of the lumen pH and a quencher (Ballottari et al., 2016; Liguori et al., 2013). At intramolecular level, the details mechanism by which excitation energy is converted into heat it still under debate, with several mechanisms proposed. Most of the different mechanisms proposed suggest that, upon NPQ induction, LHC proteins switch to a quenched state where the chlorophyll (Chl) with the lowest energy level acts as a sink of excitation energy transferring it to a nearby carotenoid (Car), populating S1 state or other carotenoid dark states where the quenching occurs (Bode et al., 2009; Liguori et al., 2017; A. V. Ruban et al., 2007). Alternatively, excitation energy has been suggested to be quenched by formation of charge transfer state between a Chl dimer (Müller et al., 2010) or through the formation of a carotenoid radical cation (Holt et al., 2005). In both cases, the formation of a quenching state requires a strong proximity between Chl and Car or between the Chl–Chl dimer. In vitro analysis on isolated and reconstituted LHC proteins was used to evaluate the role of the different chlorophyll binding sites to identify the molecular mechanisms for NPQ induction, resulting with identification of Chl 612 (Müller et al., 2010; A. V. Ruban et al., 2007), Chl 613 (Liguori et al., 2017; Z. Liu et al., 2004), or Chl 603 (Ahn et al., 2008) as the potential quenching sites, being the closest Chls to the inner Cars binding sites called L1 (Chl 612 and Chl 613) and L2 (Chl 603) and being involved in strong excitonic coupling with other Chl (Z. Liu et al., 2004; Pan et al., 2011). The identification and the characterization of binding chromophores is thus a keystone for the comprehension of quenching mechanism in LHC proteins. The quenching properties shared by different LHC proteins and the difficulties to mimic in vitro the quenching conditions where NPQ occurs, did not allow in the past to properly demonstrate which Chls bound to LHC are directly involved or not in the quenching mechanism. The intrinsic quenching properties of LHCSR, its peculiar role in NPQ induction in C. reinhardtii, and the availability of the *npq4 lhcsr1* mutant in C. reinhardtii depleted of all LHCSR subunits where no NPQ could be observed, allowing in this work to finally investigate the role of specific Chl binding sites in vivo by complementation of *npq4 lhcsr1* with LHCSR3 gene, specifically mutated on different residues responsible for Chl binding sites (Bonente et al., 2011; Liguori, Novoderezhkin, Roy, van Grondelle, & Croce, 2016).

## 2 MATERIALS AND METHODS

### 2.1 Structural model for LHCSR3

Model of the LHCSR3 structure was obtained by using CP29 structure (PDB 3PL9) (Pan et al., 2011) as a reference as described in Ballottari et al. (2016) and visualized by Chimera 1.13 software.

### 2.2 Site‐specific mutagenesis

Site‐specific mutagenesis was performed on identified Chl binding residues using QuikChange® Site‐Directed Mutagenesis Kit according to the manufacturer's instructions (Ballottari et al., 2016).

### 2.3 LHCSR3 in vitro reconstitution

LHCSR3 cDNA was cloned in pET28‐His vector for overexpression in *Escherichia coli* as reported previously (Bonente et al., 2011), removing the coding sequence for the first 14 residues—being the predicted transit peptide for chloroplast localization—which is not present in the active protein in vivo. LHCSR3 apoprotein was purified from E. coli as reported in Liguori et al. (2013) and refolded in vitro as previously described in Ballottari et al. (2016) and Bassi, Croce, Cugini, and Sandonà (1999).

### 2.4 Absorption and fluorescence steady state measurements

Absorption measurements were performed in the 350–750 nm region with a Cary 4000, Varian spectrophotometer. Steady state fluorescence measurements were performed with BeamBio custom device equipped with USB2000+ OceanOptics spectrometer and custom LED light sources for excitation.

### 2.5 Pigment analysis

Pigment analysis were performed by HPLC as described in (Lagarde, Beuf, & Vermaas, 2000). Chl a/b and Chl/Car ratios were analyzed on pigment extracts in 80% acetone by spectral deconvolution with Chl and Cars absorption forms in organic solvent as previously reported (Bassi et al., 1999; Bonente et al., 2011).

### 2.6 In vivo complementation of npq4 lhcsr1 C. reinhardtii mutant

LHCSR3.2 gene (Cre08.g367400) was amplified from C. reinhardtii genomic DNA extracted as previously described (Ballottari et al., 2016). In order to amplify the entire promoter, the region to be cloned was extended by 1000 bp at its 5′ UTR. In contrast, at 3′ UTR, a 300 bp region was selected as hypothetical terminal region. Amplified DNA was then inserted in pBC1 vector as described in Ballottari et al. (2016). *npq4 lhcsr1* mutant (Ballottari et al., 2016) was kindly gifted by Prof. Niyogi from UC Berkeley. Cells transformation and transformant selection were performed as previously described (Ballottari et al., 2016). Transformed cells were plated in TAP medium in presence of paromomycin as selective marker. Resistant colonies were then transferred in TAP medium at 25°C in flask with white light (70 μE m^−2^ s^−1^, 16 hr light/8 hr dark photoperiod). High light acclimation was induced in WT (4A+ strain), *npq4 lhcsr1* mutant and transformant lines by growing cells at 400 μE m^−2^ s^−1^ in HS medium as previously described (Ballottari et al., 2016).

### 2.7 NPQ analysis

NPQ induction curves were measured on C. reinhardtii WT, *npq4 lhcsr1* and transformant lines by using a closed‐GFPCam‐FC800 from Photon System Instruments (Czech Republic). NPQ analysis were performed on C. reinhardtii cultures in exponential phase acclimated to high light for 4 days. Measuring, saturating, and actinic light were respectively 7, 5,000, and 1,200 μmol m^−2^ s^−1^.

### 2.8 Time‐resolved fluorescence analysis

Time‐resolved fluorescence analysis was performed with a Chronos BH ISS Photon Counting instrument with picosecond laser excitation at 447 nm operating at 50 MHz. Fluorescence emissions were recorded at 685 nm in the case of isolated LHCSR complexes or at 690 nm or 715 nm in the case of whole cells, with 4 nm bandwidth. Laser power was kept below 0.1 μW; 77 K fluorescence measurements were performed on previously frozen samples. In particular, whole cells were frozen in liquid nitrogen after 20 min of dark adaptation (DARK) and after 7 min of high light treatment at 1,200 μmol m^−2^ s^−1^ (LIGHT); 77 K measurements were performed by using a cryostat (ISS) mounted on the Chronos BH ISS photon counting instrument. Fluorescence decay kinetics were analyzed with ISS Vinci 2 software by fitting with bi‐exponential or three‐exponential functions. Amplitudes (A_i_) and time constants (τ_i_) retrieved from fitting results were then used to calculate average fluorescence lifetimes (τ_avg_) as ΣA_i_τ_i_/ΣA_i_. In the case of 77 K measurements on whole cells τ_avg_ calculated for dark adapted (τ_avg DARK_) and for high light treated (τ_avg LIGHT_) samples were used to calculate the parameter 1‐(τ_avg LIGHT_/τ_avg DARK_) which is inversely proportional to the LHCSR3 quenching activity.

### 2.9 SDS‐PAGE and western blots

SDS‐PAGE was performed with a Tris‐Tricine as reported in Schägger and von Jagow (1987). Western blots were performed as reported in Bonente et al. (2011) using α‐LHCSR3 and α‐CP43 specific antibodies from Agrisera (AS14 2766 and AS11 1787, respectively).

### 2.10 Statistical analysis

All the data reported are the average of at least three biological replicates, with specific N value reported in the figure legends. Initial screening of transformant lines were performed for at least 50 positive colonies. At least five independent transformant lines were fully characterized for each LHCSR3 WT and mutant variant. Errors bars are reported as standard deviation. Linear regression reported were performed with OriginPro8.0 software. Statistical significance of linear regression was evaluated by *F* test in the “Compared dataset” function in OriginPro8.

## 3 RESULTS

### 3.1 Investigation of chlorophyll binding sites involved in NPQ in vitro

**Figure 1:**
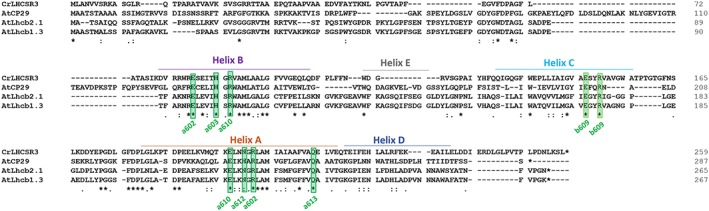
Sequence alignment of LHCSR3 with CP29 and LHCII. LHCSR3 aminoacidic sequence aligned with CP29 and LHCII (Lhcb2.1 and Lhcb1.3) sequences from Arabidopsis thaliana: conserved chlorophyll binding residues are highlighted. In the case of Chl 602, 610, and 609, the glutamate residues involved in chlorophyll binding form an ion pair with an arginine residue indicated in the alignment for each case

**Figure 2:**
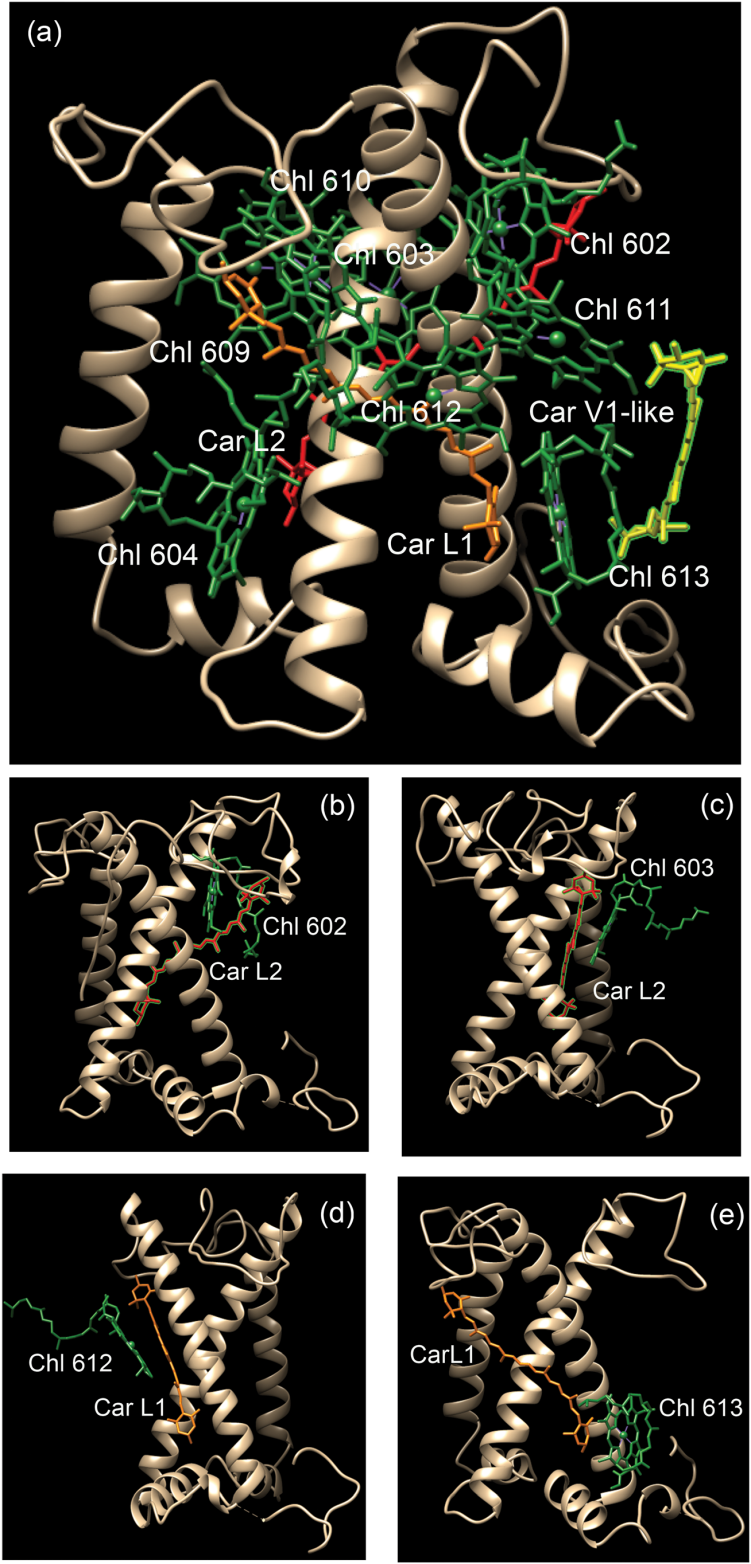
Model structure of LHCSR3. (a) Structural model of LHCSR3 obtained by sequence alignment with CP29 (PDB n. 3PL9); (b) Chl 602, (c) Chl 603, (d) Chl 612, and (e) Chl 613 in LHCSR3 model

**Figure 3:**
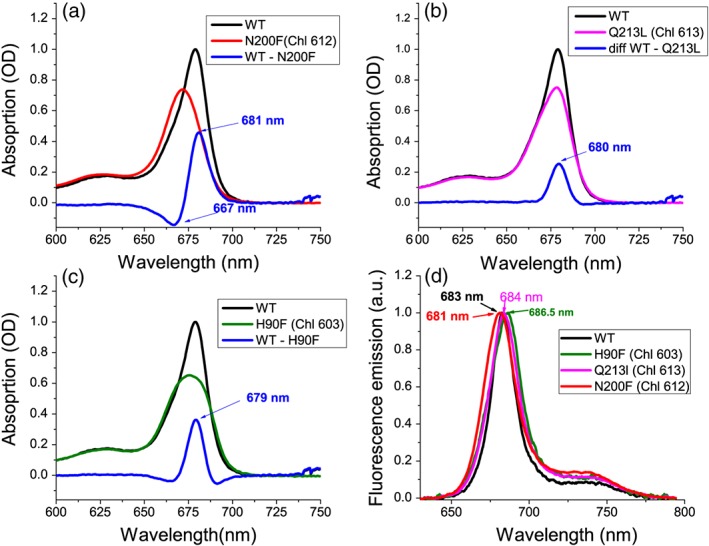
Absorption and fluorescence emission spectra of LHCSR3 WT and mutants on chlorophyll binding residues. (a–c) Absorption spectra of WT and mutants on Chl 612 (a), Chl 613 (b), and Chl 603 (c). In each case, the absorption spectra were normalized considering the chlorophyll content in each complex. Difference spectra calculated subtracting the mutant absorption spectrum to the WT spectrum are also reported. (d) Fluorescence emission spectra of LHCSR3 WT and mutants on chlorophyll binding site upon excitation at 440 nm

**Figure 4:**
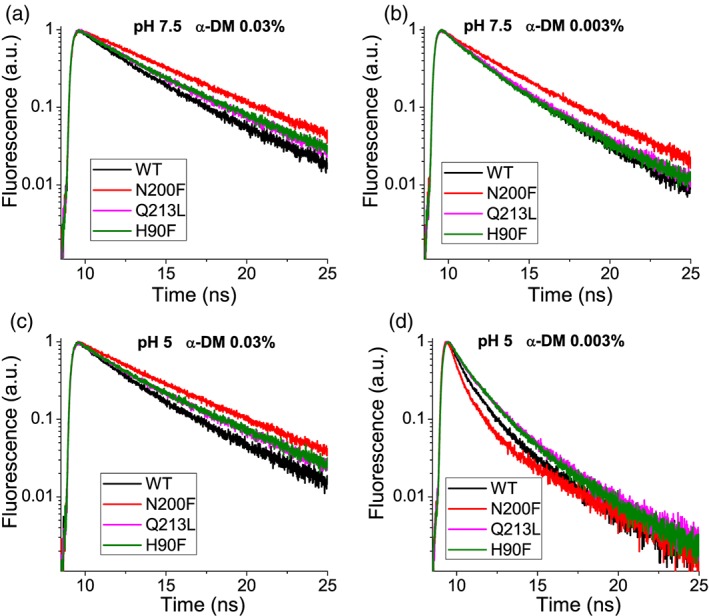
Time‐resolved fluorescence of WT and mutants on chlorophyll binding site. Fluorescence decay kinetics of WT (black), H90F (green), N200F (red), and Q213L (pink) mutants upon 447 nm excitation. Measurement were performed in Hepes buffer at pH 7.5 (a,b) or in MES buffer at pH 5 (c,d). Detergent (n‐Dodecyl α‐D‐maltoside) concentration was set at 0.03% (a,c) or at 0.003% (b,d)

Identification of potential targets for site specific mutagenesis was obtained upon sequence alignment with other LHC proteins which structure with pigments bound are available, as in particular LHCII (Z. Liu et al., 2004) and CP29 (Pan et al., 2011) (Figure 1). As reported in Figure 1, six Chl binding sites were conserved in LHCSR3 compared with LHCII and CP29, namely, residues responsible for binding Chls 602, 603, 609, 610, 612, and 613 (Bonente et al., 2011; Liguori et al., 2016). Other Chls proposed to be bound with LHCSR3 are the Chl 604, 608, and 611, which however are not coordinated by a specific residue, but rather by water or lipid molecules, making the obtainment of site‐specific mutants for these Chl binding sites impossible. Chl 612 and Chl 613 were thus chosen for mutagenesis analysis in LHCSR3 because of proximity with Car L1 site in both LHCII (Z. Liu et al., 2004) and CP29 (Pan et al., 2011), whereas chlorophylls Chl 602 and 603 were chosen being the closest Chls to Car L2 (Z. Liu et al., 2004; Pan et al., 2011; Figure 1, Table 1). In addition, Chl 612 and Chl 603 are strongly coupled with Chl 610/611 and Chl 609 and/or Chl602, respectively (Liguori et al., 2016; Z. Liu et al., 2004; Pan et al., 2011; van Amerongen & Croce, 2013). Finally, Chl 612 has been reported to be the Chl with the lowest energy level associated in most LHC protein, suggesting its potential role in quenching (van Amerongen & Croce, 2013), whereas Chl 613 was proposed to be the reddest chlorophyll in LHCSR3 (Liguori et al., 2016). Mutated version of LHCSR3 subunit on Chl binding sites were thus obtained by substituting the polar residues responsible for Chl coordination with non‐polar ones. In particular, the following residues N200, Q213, E87, and H90 responsible for Chl 612, 613, 602, and 603 binding were mutated to F, L, V, and F, respectively (Table 1), as previously reported for other LHC proteins (Ballottari, Mozzo, Croce, Morosinotto, & Bassi, 2009; Bassi et al., 1999). In the case of Chl 602, the double mutant E87V–R202L was also included in the set of LHCSR3 mutant because, as previously reported, the glutamate responsible for Chl 602 forms an ionic pair with an arginine on the opposite helix which destabilize the holoprotein if the latter is not mutated as well to a non‐charged residue (Bassi et al., 1999). LHCSR WT and mutated version were first analyzed in vitro upon protein expression in E. coli, followed by refolding with pigments to investigate the effect of the mutation introduced on the pigment binding properties of the protein complex. Among the LHCSR3 mutants reported in Table 1, either single or double mutants putatively affecting Chl 602 were not stable in vitro and no reconstituted holoproteins were obtained for these mutants. Pigment binding properties of refolded complexes were analyzed and reported in Table 2. Considering a stoichiometry of one lutein molecule per holocomplex (Bonente et al., 2011), one Chl was found to be lost in mutant complexes. In particular, one Chl a was lost in H90F mutant, whereas a small partial loss of Chl b was also detected in the case of N200F and Q213L mutants (Table 2). The loss of Chl b upon mutations on Chl binding site located close to L1 has been reported previously for other LHC complexes, as in the case of LHCB1 from Zea mays (Remelli, Varotto, Sandonà, Croce, & Bassi, 1999) or LHCB5 from Arabidopsis thaliana (Ballottari et al., 2009). In the case of mutant H90F, a strong reduction of Cars content and in particular violaxanthin was also evident: considering the preferential binding of violaxanthin to the inner Cars binding site L2 (Bonente et al., 2011), this finding suggest a partial destabilization of L2 site upon mutation to closely located Chl 603 binding site. Reconstituted holoproteins were then analyzed by absorption spectroscopy (Figure 2): absorption spectra in the visible region were normalized considering the loss of one Chl molecule in H90F, N200F, and Q213L mutant compared with WT as previously described (Bassi et al., 1999). By subtracting the mutant to WT absorption spectra (Figure 3a–c), it was possible to observe the spectral feature of the Chl lost: difference absorption spectra WT minus mutant were indeed characterized by a main peak in the 600–800 nm region at around 680 nm in all cases, confirming the predominant presence of Chl a in Chl 612, Chl 613, and Chl 603 binding site. In the case of N200F, a negative peak was also detected at 667 nm, suggesting that the mutation introduced also influenced the spectral features of other Chl in the complex, as likely the Chl 610 or Chl 611, located close to Chl 612. Fluorescence emission spectra were thus measured to determine the influence of the mutations introduced on the energy level of Chls, being the Chl with the lowest energy level the main final emitter. As reported in Figure 3d, only in the case of mutant on Chl 612 binding site (N200F) a blue shift of fluorescence emission spectra was observed, indicating that the mutation affected the final emitter. These results suggest that Chl 612 is in LHCSR3, the Chl with the lowest energy associated, as reported for other LHC proteins (van Amerongen & Croce, 2013), differently from a previous model based on excitation energy transfer measurement, which rather suggested that Chl 613 as the “reddest” chlorophyll in LHCSR3 complex (Liguori et al., 2016). Time‐resolved fluorescence lifetimes analysis was thus performed at pH 7.5 and pH 5 to investigate the pH‐dependent quenching properties of LHCSR mutants compared with WT. As reported in Figure 4, all mutants analyzed showed a slightly longer fluorescence lifetime at pH 7.5 compared with WT. A pH‐dependent quenching was then observed for all samples, decreasing the pH to 5, with a reduced quenching activity in the case of the mutant on Chl 612 binding sites. However, it is worth to note that fluorescence lifetimes of LHCSR3 complexes in high detergent conditions were in all cases higher than 1 ns (Table S1), even at pH 5, whereas in vivo, a quenching mechanism that can significantly quench the excited states of Chls should have a rate of few hundreds of ps^−1^. For this reason, time‐resolved fluorescence was also measured in WT and Chl binding sites mutants reducing the detergent concentration; in this condition proteins undergo clustering, in a condition closer to the in vivo state where these proteins are immersed in the highly crowed thylakoid membranes, with a strong reduction of fluorescence lifetime (Ballottari et al., 2016; Horton et al., 1991). At low detergent concentration, all samples were characterized by faster kinetics of fluorescence decay compared with the condition in high detergent, with a clear pH‐dependent quenching for all LHCSR3 variants. Comparing the fluorescence decay kinetics of WT and Chl binding site mutants in the most quenched conditions at low detergent concentration and pH 5, only in absence of Chl 613 and Chl 603 a reduced quenching efficiency was observed in LHCSR3 mutated holoproteins (Figure 4d), whereas in absence of Chl 612, an even stronger quenching was observed compared with the WT case.

**Table 1:** LHCSR3 mutant variants analyzed in vitro and in vivo. Mutated residues on LHCSR3 mutant variants herein investigated in vitro and in vivo are reported together with the chlorophyll binding site affected. For each chlorophyll binding site affected by mutation the closest carotenoid binding site, the distance in Å between the two conjugated π‐systems of carotenoid and Chl, as reported in the case of CP29 (Pan et al., 2011) and LHCII (Z. Liu et al., 2004) and the closest interacting chlorophylls are reported

Mutation	Chl affected	Closest Car binding site	Closest distance between the two conjugated π‐systems of carotenoid and Chl in CP29 and LHCII (Å)		Interacting chlorophylls	In vitro	In vivo
N200F	Chl 612	L1	3.73	3.65	Chl 611/CHl 610	✓	✓
Q213L	Chl 613	L1	3.83	3.88		✓	✓
E87V, E87V/R202L	Chl 602	L2	3.58	3.41	Chl 603	Not stable	Not stable
H90F	Chl 603	L2	3.7	3.7	Chl 609	✓	Not stable

**Table 2:** Pigment binding properties of recombinant LHCSR3 WT and mutant variants refolded in vitro. Pigments binding properties of LHCSR3 WT and mutant variants are reported considering eight chlorophylls bound by the following:

	Chl tot	Chl a/Chl b	Chla	Chlb	ΔChl a	ΔChl b	chl/car	Car tot	neo	viola	lutein	beta‐car
WT	7.00	7.06 ± 0.07	6.13 ± 0.06	0.87 ± 0.01	/	/	2.74 ± 0.08	2.55 ± 0.08	0.12 ± 0.17	1.49 ± 0.15	0.93 ± 0.06	0.01 ± 0.02
N200F	6.00	7.8 ± 0.17	5.32 ± 0.12	0.68 ± 0.01	0.81 ± 0.13	0.19 ± 0.02	2.61 ± 0.02	2.3 ± 0.02	0.05 ± 0.07	1.29 ± 0.16	0.94 ± 0.05	0.02 ± 0.01
Q213L	6.00	7.72 ± 0.24	5.31 ± 0.17	0.69 ± 0.02	0.82 ± 0.18	0.18 ± 0.02	2.69 ± 0.02	2.23 ± 0.02	0.1 ± 0.15	1.13 ± 0.10	0.99 ± 0.19	0.01 ± 0.01
H90F	6.00	5.94 ± 0.19	5.14 ± 0.17	0.86 ± 0.03	1 ± 0.18	0 ± 0.03	4.44 ± 0.98	1.35 ± 0.30	0.06 ± 0.08	0.39 ± 0.03	0.89 ± 0.14	0.01 ± 0.01

### 3.2 Investigation of chlorophyll binding sites involved in NPQ in vivo

**Figure 5:**
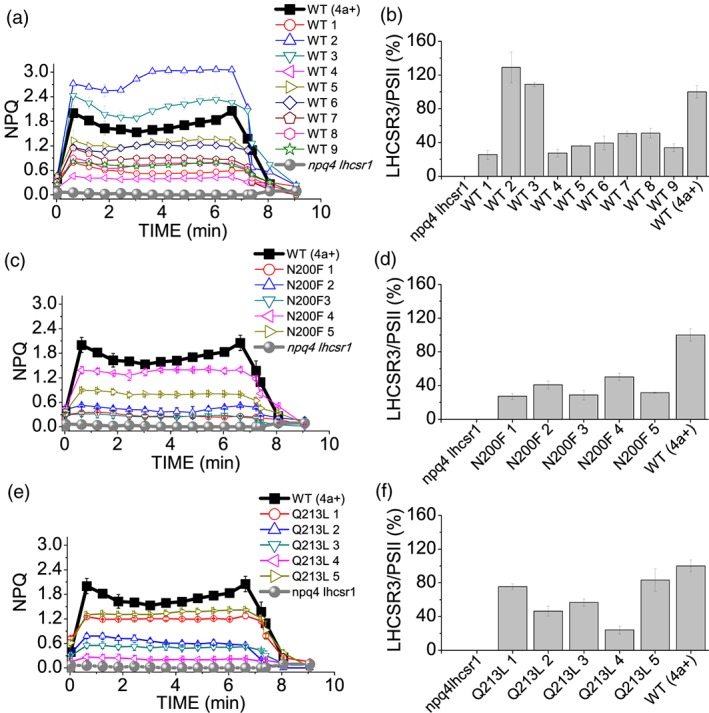
Non‐photochemical quenching (NPQ) phenotypes and accumulation of LHCSR3 in complemented lines. (a) NPQ induction kinetics and (b) LHCSR3 accumulation per PSII of Chlamydomonas reinhardtii WT (strain 4A+), npq4 lhcsr1 mutant, and transformant lines with LHCSR3.2 WT genomic sequence. (c) NPQ induction kinetics and (d) LHCSR3 accumulation per PSII of C. reinhardtii WT (strain 4A+), npq4 lhcsr1 mutant, and transformant lines with LHCSR3.2 N200F mutant variant (Chl 612 binding site). (e) NPQ induction kinetics and (f) LHCSR3 accumulation per PSII of C. reinhardtii WT (strain 4A+), npq4 lhcsr1 mutant, and transformant lines with LHCSR3.2 Q213L mutant variant (Chl 613 binding site)

**Figure 6:**
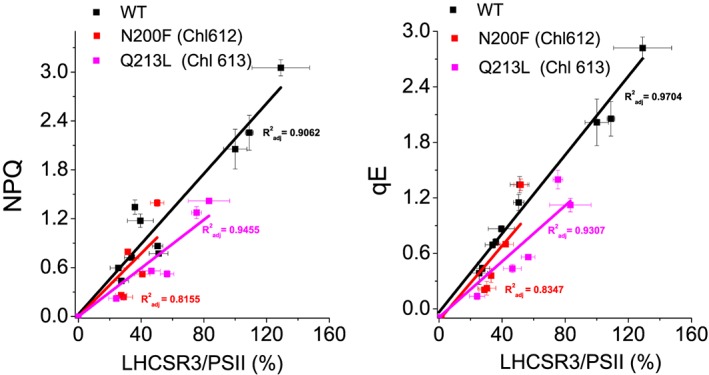
Correlation between NPQ, qE, and LHCSR3 accumulation. (a) Linear regression of non‐photochemical quenching and LHCSR3 content per PSII. (b) Linear regression of qE and LHCSR3 content per PSII. Nine and five biological independent lines were analyzed in the case of WT or mutants, respectively (three independent biological replicates for each line). Correlation coefficients are reported as adjusted R ^2^

**Figure 7:**
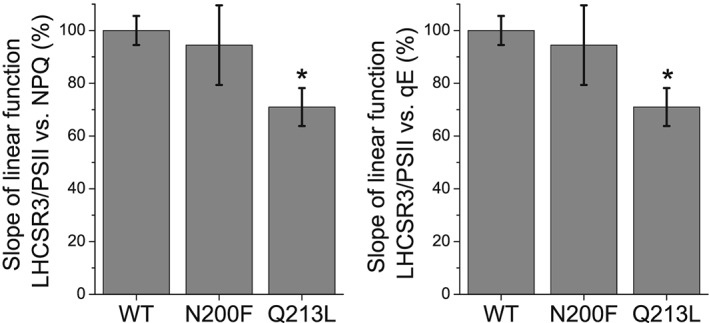
LHCSR3 quenching efficiency in WT vs. N200F and Q213L mutants. LHCSR3 quenching efficiency is reported as the slope of the linear function correlating non‐photochemical quenching (a) or qE (b) with the amount of LHCSR3 protein accumulated in each line as described in Figure 6

Because these results obtained at low detergent in vitro might be influenced by uncontrolled aggregation event caused by the site specific mutations introduced, the role of the different Chl binding residues herein investigated was then analyzed in vivo by complementation of *npq4 lhcsr1* mutant, lacking all LHCSR proteins (Ballottari et al., 2016), with WT and LHCSR3 gene mutated on Chl 612, Chl 613, Chl 602, and Chl 603 binding residues. The genomic sequence of LHCSR3, extended at 5′ UTR of 1000 bp and at 3′ of 300 bp in order to contain also the promoter and terminator regions, was inserted in the *npq4 lhcsr1* mutant. The expression of the inserted genes was thus under control of LHCSR3 endogenous promoter, which is upregulated in stressing conditions, as high light, as in the case of LHCSR3 gene in the WT background (Maruyama, Tokutsu, & Minagawa, 2014). Upon high light acclimation of transformant lines, accumulation of LHCSR3 protein and NPQ induction curves were measured. As reported in Figure S1, in the case of insertion of WT *lhcsr* gene sequence, lines with different levels of LHCSR3 were obtained. These differences are related to the different insertion sites of LHCSR3 gene, which can strongly influence gene expression. Consistently with the role of LHCSR3 in NPQ activation, complemented lines with different LHCSR3 level exhibited different kinetics of NPQ induction, with the lines with the highest LHCSR3 expression having an increased maximum NPQ induction compared with WT (Figure 5). Plotting maximum NPQ values or its rapidly reversible component qE (Rees et al., 1992), as a function of LHCSR3/PSII ratio, it was possible to observe in both cases a linear correlation between protein amount and level of energy dissipation (Figure 6a,b), as previously reported for C. reinhardtii cells acclimated to different growth conditions (Bonente, Pippa, Castellano, Bassi, & Ballottari, 2012). In the case of single mutant E87V and double mutant E87V/R502L on Chl 602 binding site, 40 resistant lines to the selection marker (paromomycin) were obtained upon transformation but none were found accumulating a detectable amount of protein despite the insertion of LHCSR3 coding genes verified by PCR ( Figure S2). These results are consistent with the destabilization of protein induced by mutation of Chl 602 binding site observed in vitro. In the case of H90F mutant on Chl 603 binding site, among the 45 transformant strains carrying the LHCSR3.2 gene, none were found to accumulate a significant amount of LHCSR3 proteins ( Figure S2). Substitution of H90 residue with F is thus likely destabilizing LHCSR3 protein in vivo*,* even if in vitro mutant H90F can be refolded upon pigment addition. As a consequence of the absence of LHCSR3 accumulation, no NPQ induction could be detected in lines transformant lines carrying E87V, E87V/R202L, or H90F LHCSR3 mutant variants. Differently, in the case of mutants on on Chl 612 (N200F) or Chl 613 (Q213L) binding site, LHCSR3 accumulation could be detected by immunoblotting upon high light acclimation (Figure S1), leading to NPQ induction (Figure 5). As in the case of WT, for both mutants on Chl 612 (N200F) or Chl 613 (Q213L) binding site, a liner correlation between maximum NPQ or qE and LHCSR3 protein quantity per PSII were observed (Figure 6c,d). In the case of N200F mutants, the slope of the linear function was almost identical compared with WT (*p* value = .62 and .57 for NPQ and qE linear correlation with LHCSR3/PSII, respectively), ruling out a possible direct involvement of Chl 612 in quenching mechanism in LHCSR3. Rather, in the case of Chl 613, a significant reduction of ~30% (Figure 7) of the slope of the liner function correlating both NPQ (*p* value = .007) or qE (*p* value = .0427) and LHCSR3/PSII was observed. These results indicate that the absence of Chl 613 reduces the quenching efficiency of the protein even if this Chl is not strictly required for NPQ induction.

**Figure 8:**
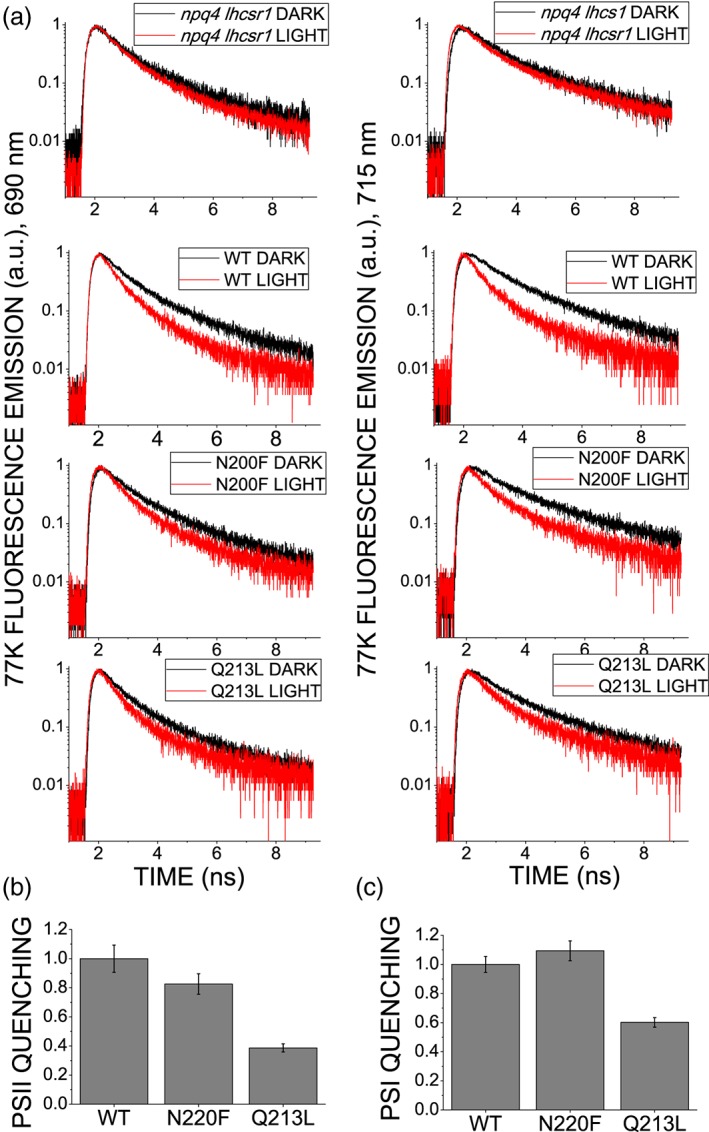
77 K chlorophyll fluorescence decay kinetics of dark adapted and high treated cells. (a) 77 K chlorophyll fluorescence decay kinetics were acquired following PSII emission at 690 nm or PSI emission at 715 nm of dark adapted (DARK) or high light treated (LIGHT, 7 min of high light treatment at 1,200 μmol m^−2^ s^−1^) cells of npq4 lhcsr1 mutant or complemented lines with lhcsr3 gene WT (line WT#7) or mutated in Chl 612 (line N200#4, accumulating 70.7% ± 5.8% LHCSR3 compared with WT#7 on a chlorophyll basis) or Chl 613 (line Q213L#3, accumulating 106% ± 5.3%% LHCSR3 compared with WT#7 on a chlorophyll basis) chlorophyll binding sites. 77 K fluorescence decay kinetics were fitted with exponential functions to calculate average fluorescence lifetime (τ_AVG_). Fitting results are reported in Table S2. (b) PSII and (c) PSI quenching calculated from τ_AVG_ of dark adapted (τ_AVG DARK_) and high light treated (τ_AVG LIGHT_) as 1‐ τ_AVG LIGHT_/τ_AVG DARK_ divided for the LHCSR3 content in each genotype on a chlorophyll basis. The resulting value was set to 1 in the case of WT. Error bars are reported as standard deviation (n = 3)

In order to further investigate the role of the different Chl binding sites in LHCSR3 quenching activity, time‐resolved fluorescence analysis was performed on whole cells before (DARK) and after (LIGHT) NPQ induction (Figure 8). Considering that chlorophyll fluorescence decay kinetics in whole cells are strongly influenced by photochemical quenching at the level of photosystems, the non‐photochemical quenching activity of *npq4 lhcsr1* lines complemented with *lhcsr3* WT, and mutant variants were investigated at 77 K in order to block photochemical reactions. Moreover, 77 K fluorescence emission enabled to discriminate between PSII and PSI emission peaks as widely reported in the literature (Nyhus, Thiel, & Pakrasi, 1993). Indeed, by measuring 77 K fluorescence decay kinetics on whole C. reinhardtii cells, LHCSR3 has been recently reported to be a quencher for both PSII supercomplexes and PSI antenna proteins (L Girolomoni et al., 2019). Fluorescence decay kinetics reported in Figure 8 demonstrate that both PSI (emission at 715 nm) and PSII (emission at 690 nm) are quenched upon NPQ induction in the lined complemented with WT or N200F and Q213L *lhcsr3* genes, but not in *npq4 lhcsr1* ( Table S2). Because the quenching observed is related to the accumulation of LHCSR3 in the cell, the reduction of the average lifetime (τ_AVG_) due to light treatment (1 ‐ τ_AVG LIGHT_/τ_AVG DARK_) was normalized to the content of LHCSR3 on a chlorophyll basis (Figure 8), revealing a ~40% decrease of both PSII and PSI fluorescence quenching efficiency in the case of Q213L mutant.

## 4 DISCUSSION

Molecular details of NPQ in LHC proteins have been strongly debated since decades: models proposed for excitation energy quenching in LHC proteins involve the formation of carotenoid radical cation (Ahn et al., 2008; Holt et al., 2005), or the population of S1 (A. V. Ruban et al., 2007) or other dark states (Liguori et al., 2017) of carotenoids, or the formation of Chl–Chl charge transfer states in strongly coupled dimers (Müller et al., 2010). Identification of Chl binding residues involved in thermal dissipation of the excitation energy is extremely important to disentangle the molecular details of NPQ. Structural details for LHCSR3 are not available yet: sequence alignment with other LHC proteins, which structure has been solved, allowed to identify the putative Chl binding residues (Bonente et al., 2011; Liguori et al., 2016). Chl 612, Chl 613, and Chl 603 are conserved in LHCSR3 (Figure S1) and were identified as the Chl closest to Car (usually lutein) in carotenoid binding site L1, in the case of Chl 612 and Chl613, or to Car in L2 (lutein in LHCII, violaxanthin in CP29) in the case of Chl 603 (Z. Liu et al., 2004; Pan et al., 2011). Moreover, these Chls have been reported to be involved in strongly coupled Chls cluster, as in particular the Chl 610‐Chl 611‐Chl 612, Chl 613‐Chl 614, and Chl 603‐609 and/or Chl 603‐602 clusters (Liguori et al., 2016; Z. Liu et al., 2004; Pan et al., 2011). For these reasons, any quenching mechanisms proposed up to now suggested a key role for one of these Chls (Ahn et al., 2008; Ballottari, Girardon, Betterle, Morosinotto, & Bassi, 2010; Kim et al., 2017; Liguori et al., 2016; Z. Liu et al., 2004; A. V. Ruban et al., 2007). The involvement of Chl 612, Chl 613, Chl 602, and Chl 603 in quenching properties of LHCSR3 was thus evaluated in this work in vitro and in vivo. In vitro spectroscopic analysis of LHCSR3 mutants on residues responsible for binding the aforementioned Chls demonstrated the presence of essentially Chl a in the Chl binding residue herein investigated, among which Chl 612 resulted to be the Chl with the lowest energy level associated, a conservative trait at least among LHC proteins constituting the peripheral antenna system of PSII (van Amerongen & Croce, 2013). Loss of Chl 612 clearly affected the final emitter of the complex (Figure 3d) but measuring fluorescence lifetime in “quenching” conditions induced by aggregation state at low pH, mutant on Chl 612 was even more quenched compared with the WT (Figure 4d). Because fluorescence decay kinetics in vitro at low detergent conditions might be influenced by possible aggregation dependent side effects due to the mutation introduced, in vivo quenching activity of LHCSR3 Chl binding sites were then studied in vivo. Mutation on Chl 612 binding site in vivo clearly demonstrates that, correlating NPQ and LHCSR3 protein level, in absence of Chl 612 the induction of NPQ was similar or even slightly stronger compared with the WT case, in agreement with the in vitro results. These results suggest that Chl 612 is not directly involved in NPQ induction in vivo in LHCSR3. Differently, in the case of Chl 613, a reduction of specific LHCSR quenching activity was evident (Figures 4, 6–8), even if the loss of this Chl did not completely impair NPQ induction, in agreement with quenching analysis in vitro. Chl 613 binding site can thus be identified as a protein domain with a peculiar, but not exclusive, role in the quenching activity of LHCSR3 proteins. In the case of Chl 613, its location close to Car in L1 (Table 1) makes this Chl‐Car interaction site suitable for energy transfer to Car S1 or other dark state. The absence of Chl 614 in LHCSR3 (Liguori et al., 2016), being this Chl the sole Chl excitonically coupled with Chl 613 in other LHC proteins, rules out a possible Chl–Chl charge transfer state (Müller et al., 2010) as NPQ mechanism at Chl 613 site. Alternatively, the finding of LHCSR3 as a dimer attached to PSII supercomplex might suggest that Chl 613 interact with another Chl of the interacting monomer forming a potential Chl–Chl charge transfer site. By the way, the reduced but still significant quenching activity observed in LHCSR3 mutated on Chl 613 strongly suggest that indeed multiple quenching sites are present, which cooperatively dissipate the excitation energy. In the case of Car in L2, it was not possible to yield information in vivo on the role of Chl 602 or Chl 603, the closest Chls to this Car, because of impaired protein accumulation in vivo in this mutant. In this case, a possible structural role of Chl binding site located close to Car in L2 can be proposed for LHCSR3 homocomplex formation. Indeed, in the case of Chl 603 mutant a strong destabilization of L2 Cars binding site was evident (Table 2). It should also be noted that Cars in L2 site, Chl 602 and Chl 603 have been reported to be the main quenching site of Chl triplets (Ballottari, Mozzo, Girardon, Hienerwadel, & Bassi, 2013), with a strong increase of ROS formation in vitro in mutants variants of LHC proteins depleted of this Chls. Upon high light exposure of transformant lines, for inducing protein accumulation, this mutated version of LHCSR3 could be rapidly degraded because of the generation of a high level of unquenched Chl triplets, and consequently ROS. The quenching efficiency of LHCSR3 mutated on Chl 603 was anyway investigated in vitro, observing a similar behavior compared with mutant on Chl 613 binding site. These results suggest that protein domain formed by Car in L2 and Chl 603 or Chl 602 is likely a quenching site, which cooperatively acts with other quenching sites, as Chl 613 and Car in L1 dimer, to dissipate excitation energy trapped by LHCSR3 protein. Chl 603 could be involved in energy transfer to Car in L2, or in formation of Car radical cation, as previously reported in the case of other LHC proteins (Ahn et al., 2008; Ballottari et al., 2014), or even in formation of Chl‐Chl charge transfer state. Finally, by measuring time‐resolved fluorescence at 77 K in whole cells before and after NPQ induction, it was possible to investigate LHCSR3‐dependent quenching not only on PSII fluorescence but also on fluorescence deriving from PSI (Figure 8). LHCSR3 dependent quenching of PSI fluorescence has been recently reported to be independent from state transitions activation and STT7 activity but specifically occurring at the level of LHC complexes bound to PSI (L Girolomoni et al., 2019). The results obtained and reported in Figure 8 demonstrates that PSI quenching is again affected, considering the LHCSR3 protein content, upon mutation on Chl 613 but not upon mutation on Chl 612.

In conclusion, this works allowed to elucidate the molecular mechanisms for NPQ in LHCSR3 in C. reinhardtii, which consist into multiple quenching sites that cooperatively dissipate the excitation energy harvested directly by LHCSR3 or received by other interacting chlorophyll‐binding proteins (Elrad, Niyogi, & Grossman, 2002; L. Girolomoni et al., 2017; Semchonok et al., 2017). A further hypothesis is that LHCSR3 might not be the direct quencher of excitation energy, but rather a pH‐dependent trigger switching other LHC protein to a quenched state, as previously suggested in the case of PSBS in higher plants (Correa‐Galvis et al., 2016). These results can thus be applied in engineering NPQ process to improve photosynthetic efficiency and biomass production properly tuning photoprotection properties of plant cells.

## AUTHOR CONTRIBUTIONS

M.B. conceived the work and wrote the paper. F.P. performed all the experiments reported. S.C. contributed to the selection and characterization of transformant strains and to the 77 K time‐resolved fluorescence measurements. All authors analyzed the results, contributed to writing, and approved the final version of the manuscript. M.B. agrees to serve as the author responsible for contact and ensures communication.

## Supporting information


**Table S1:** **Time resolved fluorescence analysis and average fluorescence decay lifetimes of LHCSR3 WT and mutant proteins refolded in vitro.** Decay kinetics reported in the main text in Figure 4 were fitted with a three‐exponential decay function Vinci 2 software from ISS. Amplitudes (Ai) and time constants (τi) are reported. Average fluorescence lifetimes (τavg) were calculated as ΣAiτi/ΣAi. Standard deviations are <5% (n = 3).
**Table S2: 77 K time resolved fluorescence analysis and average fluorescence decay lifetimes of LHCSR3 WT and mutant proteins refolded in vitro.** High light acclimated npq4 lhcsr1 cells and complemented lines with LHCSR3 WT or mutants on Chl 612 (N220F) or Chl 603 (Q213L) were frozen in liquid nitrogen after 20 minutes of dark adaptation (DARK) and after 7 minutes of high light treatment at 1200 μmol m‐2 s‐1 (LIGHT). 77 K fluorescence emission were then measured at 690 nm or 715 nm in order to follow PSII or PSI fluorescence respectively. Fluorescence kinetics were then fitted with a bi‐exponential decay function Vinci 2 software from ISS: amplitudes (Ai) and time constants (τi) are reported. Average fluorescence lifetimes (τavg) were calculated as ΣAiτi/ΣAi. τavg calculated for dark adapted (τavg DARK) and for high light treated (τavg LIGHT) samples were then used to calculate the parameter 1‐(τavg LIGHT/τavg DARK) which is inversely proportional to the LHCSR3 quenching activity. Standard deviations are <10% (n = 3).
**Figure S1: Western blot analysis of LHCSR3 accumulation upon npq4 lhcsr1 complementation.** Western blot analysis of LHCSR3 accumulation upon npq4 lhcsr1 complementation with LHCSR3.2 WT gene sequence (**A**) or mutated on CHl 612 (**B**) or 613 (**C**) chlorophyll binding sites. Sample were loaded in three replicates at different amount of chlorophylls. μg of loaded chlorophylls are indicated. Western blots were developed using antibodies specific for LHCSR3 and CP43, the latter as a marker for PSII**.**
**Figure S2: npq4 lhcsr1 transformation with H90F and H87V‐R202L LHCSR3.2 mutant variant.** The presence of genes coding for LHCR3 and resistance for paromomycin (AphVIII) in transformant strains were tested by PCR on genomic DNA extracted from colonies grown in selective plates. pBC1 vector used for transformation was used as positive control while genomic DNA extracted from npq4 lhcsr1 untransformed strain was used as negative control. Western blot on total protein extract is reported on the bottom, with WT 4A+ as positive control. Three independent lines for each mutation are reported as representative of the different resistant lines screened. 40 resistant lines to the selection marker (paromomycin) were obtained and verified for the presence of LHCSR3 coding sequence in the case of each mutant E87V and E87V‐R502L on Chl 602 binding site while 45 transformant strains were obtained and verified in the case of H90F mutant on Chl 603 chlorophyll binding site.
