# Supplementary material for: In vitro and in vivo investigation of chlorophyll binding sites involved in non‐photochemical quenching in Chlamydomonas reinhardtii
Source: Plant Cell Environ. 2019 May 9;42(8):2522–35. doi: 10.1111/pce.13566 (PMC6767442; doi:10.1111/pce.13566)

**SUPPLEMENTARY INFORMATION**

**Table S1: Time resolved fluorescence analysis and average fluorescence decay lifetimes of LHCSR3 WT and mutant proteins refolded *in vitro*.** Decay kinetics reported in the main text in Figure 4 were fitted with a three-exponential decay function Vinci 2 software from ISS. Amplitudes ($A_i$) and time constants ($\tau_i$) are reported. Average fluorescence lifetimes ($\tau_{avg}$) were calculated as $\Sigma A_i\tau_i/\Sigma A_i$. Standard deviations are < 5% (n=3).

| | $A_1$ | $\tau_1$ (ns) | $A_2$ | $\tau_2$ (ns) | $A_3$ | $\tau_3$ (ns) | $\tau_{avg}$ (ns) |
|---|---|---|---|---|---|---|---|
| **WT pH7.5 0,03% α-DM** | 0.44 | 4.35 | 0.51 | 1.87 | 0.04 | 0.53 | 2.91 |
| **WT pH 5 0,03% α-DM** | 0.37 | 4.42 | 0.50 | 1.83 | 0.13 | 0.40 | 2.59 |
| **WT pH7.5 0,003% α-DM** | 0.32 | 3.91 | 0.55 | 1.91 | 0.14 | 0.45 | 2.34 |
| **WT pH 5 0,003% α-DM** | 0.09 | 3.12 | 0.52 | 0.92 | 0.39 | 0.23 | 0.83 |
| | | | | | | | |
| **N220F pH7.5 0,03% α-DM** | 0.84 | 4.87 | 0.13 | 1.38 | 0.03 | 0.50 | 4.30 |
| **N220F pH 5 0,03% α-DM** | 0.70 | 4.92 | 0.23 | 1.57 | 0.07 | 0.35 | 3.83 |
| **N220F pH7.5 0,003% α-DM** | 0.53 | 4.31 | 0.28 | 1.82 | 0.19 | 0.33 | 2.87 |
| **N220F pH 5 0,003% α-DM** | 0.05 | 3.68 | 0.42 | 0.76 | 0.54 | 0.24 | 0.62 |
| | | | | | | | |
| **Q213L pH7.5 0,03% α-DM** | 0.54 | 4.76 | 0.41 | 1.81 | 0.06 | 0.25 | 3.31 |
| **Q213L pH 5 0,03% α-DM** | 0.51 | 4.75 | 0.41 | 1.65 | 0.08 | 0.35 | 3.12 |
| **Q213L pH7,5 0,003% α-DM** | 0.31 | 4.26 | 0.54 | 1.87 | 0.15 | 0.33 | 2.37 |
| **Q213L pH 5 0,003% α-DM** | 0.10 | 3.51 | 0.47 | 1.31 | 0.43 | 0.37 | 1.13 |
| | | | | | | | |
| **H90F pH7,5 0,03% α-DM** | 0.58 | 4.82 | 0.38 | 1.75 | 0.04 | 0.48 | 3.47 |
| **H90F pH 5 0,03% α-DM** | 0.50 | 4.78 | 0.38 | 1.65 | 0.11 | 0.41 | 3.09 |
| **H90F pH7,5 0,003% α-DM** | 0.28 | 4.30 | 0.56 | 1.79 | 0.16 | 0.40 | 2.27 |
| **H90F pH 5 0,003% α-DM** | 0.10 | 3.48 | 0.54 | 1.30 | 0.36 | 0.38 | 1.18 |

**Table S2: 77K time resolved fluorescence analysis and average fluorescence decay lifetimes of LHCSR3 WT and mutant proteins refolded *in vitro.*** High light acclimated *npq4 lhcsr1* cells and complemented lines with LHCSR3 WT or mutants on Chl 612 (N220F) or Chl 603 (Q213L) were frozen in liquid nitrogen after 20 minutes of dark adaptation (DARK) and after 7 minutes of high light treatment at 1200 µmol $m^{-2}$ $s^{-1}$ (LIGHT). 77K fluorescence emission were then measured at 690 nm or 715 nm in order to follow PSII or PSI fluorescence respectively. Fluorescence kinetics were then fitted with a bi-exponential decay function Vinci 2 software from ISS: amplitudes ($A_i$) and time constants ($\tau_i$) are reported. Average fluorescence lifetimes ($\tau_{avg}$) were calculated as $\Sigma A_i\tau_i/\Sigma A_i$. $\tau_{avg}$ calculated for dark adapted ($\tau_{avg\ DARK}$) and for high light treated ($\tau_{avg\ LIGHT}$) samples were then used to calculate the parameter 1-($\tau_{avg\ LIGHT}$ / $\tau_{avg\ DARK}$) which is inversely proportional to the LHCSR3 quenching activity. Standard deviations are < 10% (n=3).

| PSII emission (690 nm) | | $A_1$ | $\tau_1$ (ns) | $A_2$ | $\tau_2$ (ns) | $\tau_{avg}$ (ns) | 1-($\tau_{avg\ LIGHT}$ / $\tau_{avg\ DARK}$) |
|---|---|---|---|---|---|---|---|
| **WT #7** | **DARK** | 0.50 | 2.37 | 0.5 | 0.55 | 1.46 | 0.56 |
| | **LIGHT** | 0.40 | 1.53 | 0.603 | 0.35 | 0.8 | |
| **N200F #4** | **DARK** | 0.56 | 2.37 | 0.443 | 0.58 | 1.58 | 0.66 |
| | **LIGHT** | 0.46 | 1.78 | 0.54 | 0.41 | 1.04 | |
| **Q213L #3** | **DARK** | 0.50 | 2.25 | 0.499 | 0.46 | 1.35 | 0.72 |
| | **LIGHT** | 0.36 | 2.04 | 0.644 | 0.38 | 0.97 | |
| ***npq4 lhcsr1*** | **DARK** | 0.51 | 2.26 | 0.488 | 0.56 | 1.43 | 0.95 |
| | **LIGHT** | 0.37 | 2.66 | 0.629 | 0.61 | 1.37 | |
| | | | | | | | |
| **PSII emission (715 nm)** | | **$A_1$** | **$\tau_1$ (ns)** | **$A_2$** | **$\tau_2$ (ns)** | **$\tau_{avg}$ (ns)** | **1-($\tau_{avg\ LIGHT}$ / $\tau_{avg\ DARK}$)** |
| **WT #7** | **DARK** | 0.56 | 2.81 | 0.44 | 0.79 | 1.93 | 0.52 |
| | **LIGHT** | 0.53 | 1.61 | 0.47 | 0.31 | 0.999 | |
| **N200F #4** | **DARK** | 0.69 | 2.94 | 0.31 | 0.75 | 2.25 | 0.61 |
| | **LIGHT** | 0.58 | 2.07 | 0.42 | 0.40 | 1.37 | |
| **Q213L #3** | **DARK** | 0.64 | 2.62 | 0.36 | 0.69 | 1.93 | 0.77 |
| | **LIGHT** | 0.49 | 2.49 | 0.51 | 0.52 | 1.49 | |
| ***npq4 lhcsr1*** | **DARK** | 0.56 | 2.74 | 0.44 | 0.76 | 1.88 | 0.89 |
| | **LIGHT** | 0.60 | 2.44 | 0.40 | 0.52 | 1.68 | |

**Figure S1: Western blot analysis of LHCSR3 accumulation upon npq4 lhcsr1 complementation.** Western blot analysis of LHCSR3 accumulation upon *npq4 lhcsr1* complementation with LHCSR3.2 WT

gene sequence (**A**) or mutated on CHl 612 (**B**) or 613 (**C**) chlorophyll binding sites. Sample were loaded in three replicates at different amount of chlorophylls. μg of loaded chlorophylls are indicated. Western blots were developed using antibodies specific for LHCSR3 and CP43, the latter as a marker for PSII**.**

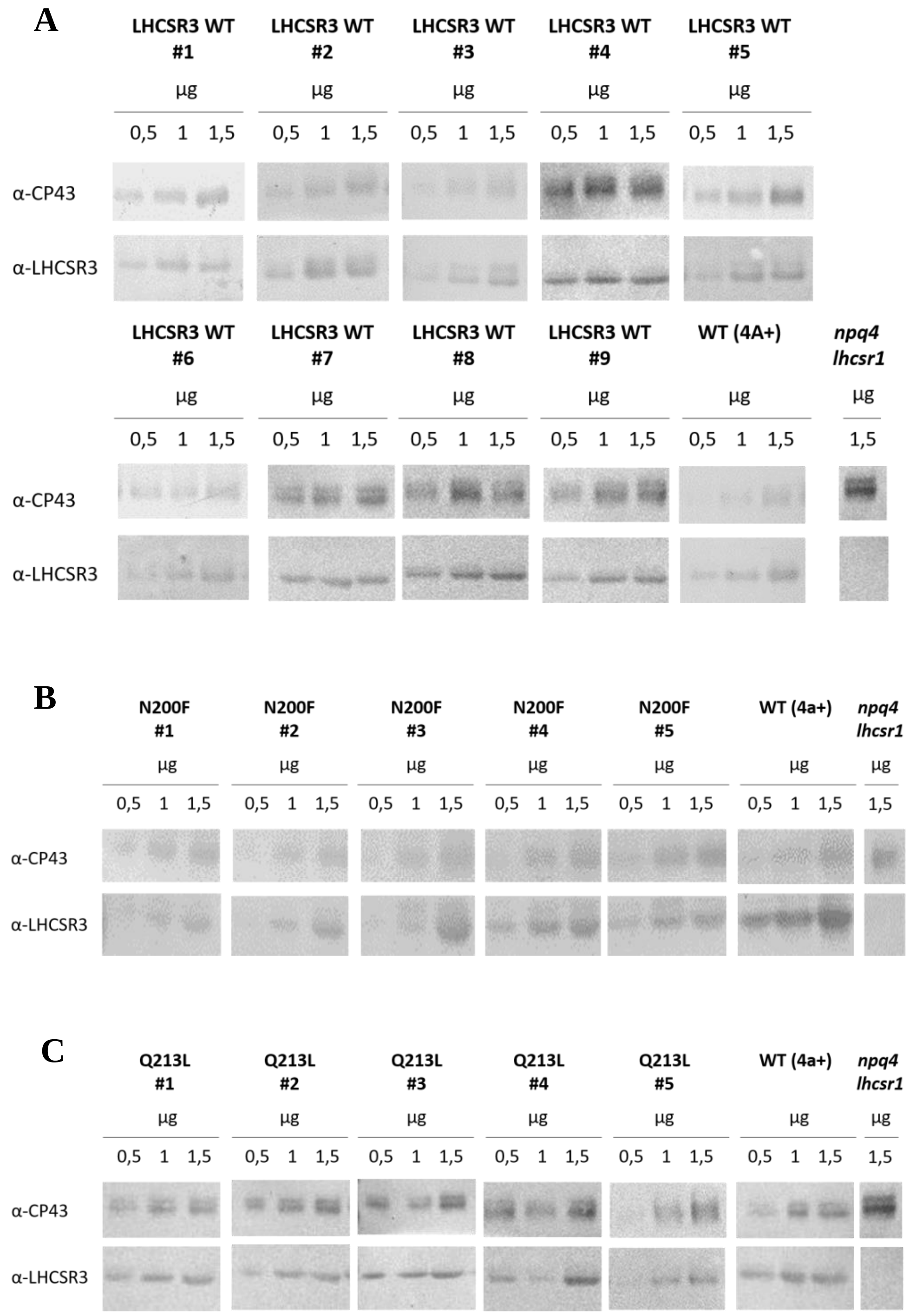


**Figure S2: *npq4 lhcsr1* transformation with H90F and H87V-R202L LHCSR3.2 mutant variant.** The presence of genes coding for LHCR3 and resistance for paromomycin (AphVIII) in transformant strains were tested by PCR on genomic DNA extracted from colonies grown in selective plates. pBC1 vector used

for transformation was used as positive control while genomic DNA extracted from *npq4 lhcsr1* untransformed strain was used as negative control. Western blot on total protein extract is reported on the bottom, with WT 4A+ as positive control. Three independent lines for each mutation are reported as representative of the different resistant lines screened. 40 resistant lines to the selection marker (paromomycin) were obtained and verified for the presence of LHCSR3 coding sequence in the case of each mutant E87V and E87V-R502L on Chl 602 binding site while 45 transformant strains were obtained and verified in the case of H90F mutant on Chl 603 chlorophyll binding site.

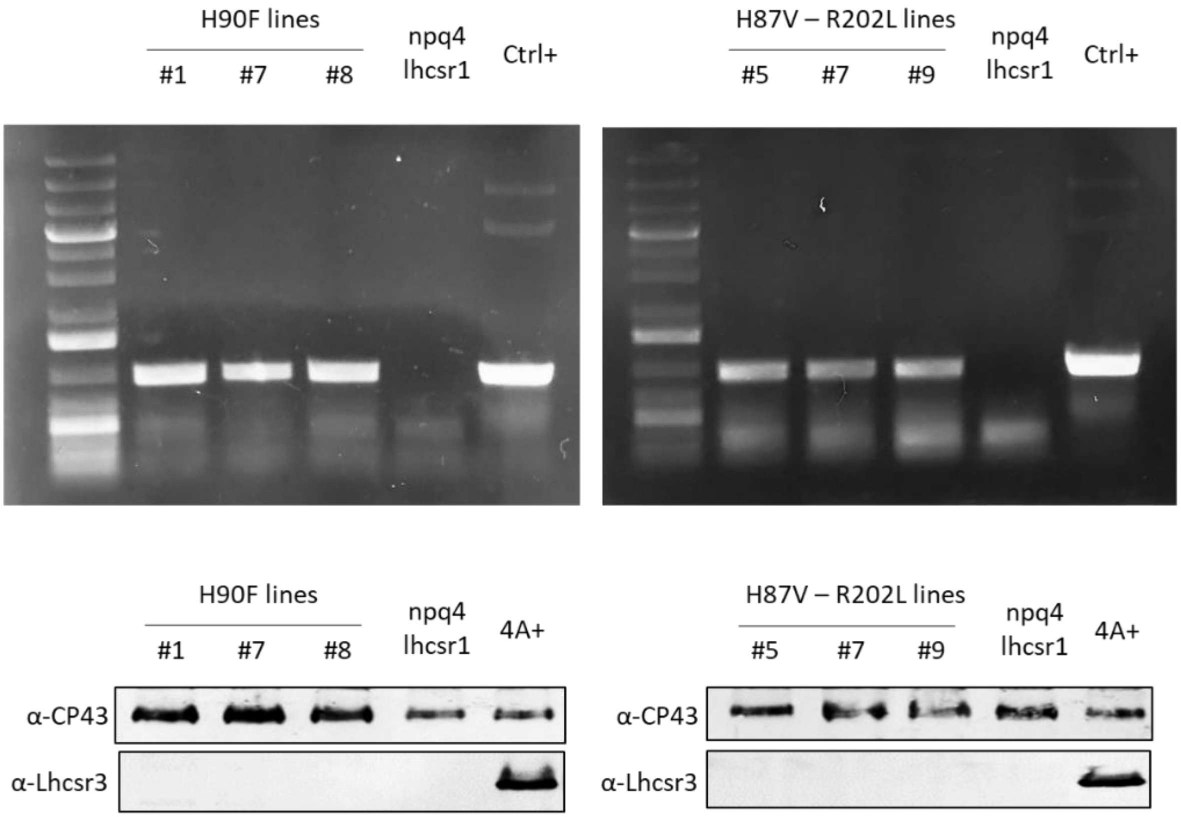

Supplement: Supplementary file 1 — Table S1: Time resolved fluorescence analysis and average fluorescence decay lifetimes of LHCSR3 WT and mutant proteins refolded in vitro. Decay kinetics reported in the main text in Figure 4 were fitted with a three‐exponential decay function Vinci 2 software from ISS. Amplitudes (Ai) and time constants (τi) are reported. Average fluorescence lifetimes (τavg) were calculated as ΣAiτi/ΣAi. Standard deviations are <5% (n = 3). Table S2: 77 K time resolved fluorescence analysis and average fluorescence decay lifetimes of LHCSR3 WT and mutant proteins refolded in vitro. High light acclimated npq4 lhcsr1 cells and complemented lines with LHCSR3 WT or mutants on Chl 612 (N220F) or Chl 603 (Q213L) were frozen in liquid nitrogen after 20 minutes of dark adaptation (DARK) and after 7 minutes of high light treatment at 1200 μmol m‐2 s‐1 (LIGHT). 77 K fluorescence emission were then measured at 690 nm or 715 nm in order to follow PSII or PSI fluorescence respectively. Fluorescence kinetics were then fitted with a bi‐exponential decay function Vinci 2 software from ISS: amplitudes (Ai) and time constants (τi) are reported. Average fluorescence lifetimes (τavg) were calculated as ΣAiτi/ΣAi. τavg calculated for dark adapted (τavg DARK) and for high light treated (τavg LIGHT) samples were then used to calculate the parameter 1‐(τavg LIGHT/τavg DARK) which is inversely proportional to the LHCSR3 quenching activity. Standard deviations are <10% (n = 3). Figure S1: Western blot analysis of LHCSR3 accumulation upon npq4 lhcsr1 complementation. Western blot analysis of LHCSR3 accumulation upon npq4 lhcsr1 complementation with LHCSR3.2 WT gene sequence (A) or mutated on CHl 612 (B) or 613 (C) chlorophyll binding sites. Sample were loaded in three replicates at different amount of chlorophylls. μg of loaded chlorophylls are indicated. Western blots were developed using antibodies specific for LHCSR3 and CP43, the latter as a marker for PSII. Figure S2: npq4 lhcsr1 transformation with H9 [file PCE-42-2522-s001.pdf]
